# Tissue Engineering in Animal Models for Urinary Diversion: A Systematic Review

**DOI:** 10.1371/journal.pone.0098734

**Published:** 2014-06-25

**Authors:** Marije Sloff, Rob de Vries, Paul Geutjes, Joanna IntHout, Merel Ritskes-Hoitinga, Egbert Oosterwijk, Wout Feitz

**Affiliations:** 1 Department of Urology, Nijmegen Centre for Molecular Life Sciences, Radboud University Medical Center, Nijmegen, The Netherlands; 2 SYRCLE (SYstematic Review Centre for Laboratory animal experimentation), Central Animal Laboratory, Radboud University Medical Center, Nijmegen, The Netherlands; 3 Department for Health Evidence, Radboud University Medical Center, Nijmegen, The Netherlands; University of California, Merced, United States of America

## Abstract

Tissue engineering and regenerative medicine (TERM) approaches may provide alternatives for gastrointestinal tissue in urinary diversion. To continue to clinically translatable studies, TERM alternatives need to be evaluated in (large) controlled and standardized animal studies. Here, we investigated all evidence for the efficacy of tissue engineered constructs in animal models for urinary diversion. Studies investigating this subject were identified through a systematic search of three different databases (PubMed, Embase and Web of Science). From each study, animal characteristics, study characteristics and experimental outcomes for meta-analyses were tabulated. Furthermore, the reporting of items vital for study replication was assessed. The retrieved studies (8 in total) showed extreme heterogeneity in study design, including animal models, biomaterials and type of urinary diversion. All studies were feasibility studies, indicating the novelty of this field. None of the studies included appropriate control groups, i.e. a comparison with the classical treatment using GI tissue. The meta-analysis showed a trend towards successful experimentation in larger animals although no specific animal species could be identified as the most suitable model. Larger animals appear to allow a better translation to the human situation, with respect to anatomy and surgical approaches. It was unclear whether the use of cells benefits the formation of a neo urinary conduit. The reporting of the methodology and data according to standardized guidelines was insufficient and should be improved to increase the value of such publications. In conclusion, animal models in the field of TERM for urinary diversion have probably been chosen for reasons other than their predictive value. Controlled and comparative long term animal studies, with adequate methodological reporting are needed to proceed to clinical translatable studies. This will aid in good quality research with the reduction in the use of animals and an increase in empirical evidence of biomedical research.

## Introduction

Urinary diversion with gastrointestinal (GI) tissue remains the gold standard treatment for patients suffering from end-stage bladder disease caused by bladder cancer or congenital malformations, e.g. bladder exstrophy or spina bifida [1], [2]. There are three approaches to create a urinary diversion in these patients. The first and most commonly used type among surgeons is the incontinent ileocutaneostomy; a urinary conduit with a skin-outlet. Alternatively, continent diversions can be formed non-orthotopically with a skin-outlet or orthotopically as a neobladder [3], [4]. Although the use of GI tissue provides a satisfactory outcome in most cases, it can be associated with severe complications. These can be either related to the bowel surgery (obstruction, infections, fistulas, etc.) or to the urostomy implantation (metabolic disorders, stone formations, infections, etc.) [5], [6].

A tissue engineering and regenerative medicine (TERM) approach may provide new possibilities by creating a man-made construct to replace GI tissue for urinary diversions. The implementation of such constructs could prevent invasive bowel surgery and the potentially life-threatening complications, therefore reducing health care costs. Several investigators have focused on the development of new materials for this purpose, including naturally derived materials, synthetic polymers and decellularized scaffolds [7]–[9]. These biomaterials can be applied with and without autologous cells, using the regenerative capacity of the body [10], [11].

In the field of urogenital reconstruction, bladder domes for cystoplasty and uretheral reconstruction with man-made constructs have already been used in patients [12], [13]. However, despite the progress in *in vitro* research and animal experimentation, clinical translation of TERM approaches for urinary diversion has been negligible. Translation from bench to bedside for these tissue engineered constructs starts with the analysis of biodegradability, biocompatibility and foreign body response, which is usually performed in small rodents. To engineer and regenerate specific tissues, evaluation should preferably be performed at relevant anatomical sites with appropriately sized constructs to permit easy clinical translation. Large animal models closely mimicking the human body are therefore desirable, but their use might be ethically debatable [14]. In general, the choice of an animal model is dependent on financial considerations, the investigators experience, ethical sensitivity and practical limitations. Even though other and better translatable models might be available [15]. To our knowledge a superior animal for tissue engineering and urinary diversion has not been identified yet.

To decide on the most suitable type of animal model an evidenced-based systematic review is essential, since it potentially increases the chance of successful clinical translation [16], [17]. We therefore systematically searched the current literature for all types of studies on the efficacy of tissue engineered constructs in animal models for urinary diversion. The results were analyzed with respect to survival, side effects, functionality and urothelium formation, to investigate whether there was sufficient evidence to decide if any animal model was superior for evaluating tissue engineered constructs for urinary diversion applications.

## Materials and Methods

### 1. Search strategy

We identified relevant studies, including peer reviewed articles and (congress) abstracts, through a systematic search of PubMed, EMBASE (OvidSP) and Web of Science up until the 23^rd^ of January 2013, following the approach as described by de Vries et al., and Leenaars et al. [16], [17]. In all three databases synonyms for tissue engineering (e.g. tissue engineering, tissue engineered, regenerative medicine or biomaterials) were combined with synonyms for urinary diversion (e.g. orthotopic diversion, neobladders, continent or incontinent stomas). MeSH terms (PubMed) and EMTREE terms (EMBASE) were used when available and were combined with additional free-text words from titles or abstracts ([tiab] or/ti,ab.). For the complete strategy, see Table S1. In PubMed and Embase (OvidSP), the results were filtered for animal studies, using previously designed ‘animal filters’ [18], [19]. The included primary studies and relevant reviews on the subject were screened for additional relevant references.

### 2. Study selection

Only primary studies that evaluated tissue engineered construct for urinary diversion in animal models were included. From the retrieved set of papers, duplicates and triplicates were manually deleted from EndNote, considering the preference PubMed>EMBASE>Web of Science. Based on title/abstract, primary screening was performed by a single review author (MS), deleting articles that clearly did not involve tissue engineering or urinary diversion. In case of any doubt, articles were included for further screening. Secondary screening of title/abstract was independently performed in Early Review Organizing Software (EROS, IECS, Buenos Aires, Argentina, www.eros-systematic-review.org) by two review authors (MS and RdV). The following inclusion criteria were used: 1) urinary diversion, 2) tissue engineering, 3) (living) animals of any species, and 4) primary articles. In this step, a procedure was considered to be a urinary diversion if it involved a total/radical cystectomy or implantation of a stoma or pouch connected to at least one ureter. Articles that described a partial cystectomy, hemi-cystectomy or bladder augmentation were excluded, because they do not relate to urostomy. Tissue engineered constructs were defined as biomaterials or polymers that aided the (re)construction of tissues. Articles were either categorized as ‘included’, ‘excluded’ or ‘more information necessary’ if important details were not included in the abstract. Any discrepancies were discussed and re-evaluated until consensus was reached. Full-text articles were retrieved and evaluated for definite inclusion/exclusion, based on the same criteria used for the secondary screening. The reference lists of the included studies and manually identified reviews on the subject were screened for any missed references. Unfortunately, one of the included studies was published in Korean and we did not have the resources to have it translated [20]. The article was therefore excluded from this review.

### 3. Data extraction

From every included study, basic information (author, year of publication, etc.), animal characteristics (species, sex, etc) and study characteristics (biomaterial, follow-up, etc.) were extracted and tabulated by MS and RdV after reaching consensus (Table 1). The outcome of the studies for the meta-analysis was assessed using extracted data on mortality, adverse effects, occlusion (blockade of urinary flow) and the formation of urothelium on the implanted construct (Table 2).

**Table 1:** Study characteristics.

	Reference	Publication	Species (strain)	Sex	weight/ age	Group size	Type of intervention	Biomaterial	Size scaffold	Cell type/ amount	Culture period	Study Design	Evaluation time point	outcome measures
1	Basu 2012	Research paper	Minipigs (Göttingen swine)	M/F	12–16 kg	gr 1: 8 gr 2: 8 gr 3: 8 gr 4: 8	Urinary conduit 2 ureters	PLGA-coated PGA	*	1 cm2 bladder biopsy, 2 cm2 adipose biopsy 50 mL peripheral blood All: 30–40×10∧6 SMC	6 days	gr 1: bladder SMC gr 2: adipose SMC gr 3: blood SMC gr 4: unseeded	84+/−5 days	Occlusion? Histology Immunohistochemistry
2	Bertram 2009	Poster	Canines	*	*	gr 1: 8 gr 2: 8 gr 3: 8 gr 4: 8	Neo-bladder	Tengion Autologous Neo-bladder (PLGA)	*	SMC	*	gr 1: 4×10∧6 SMC gr 2: 12×10∧6 SMC gr 3: 25×10∧6 SMC gr 4: reimplanted bladder	9 months	Histology Contractile response Electrical field stimulation
3	De Filippo 2009	Abstract	*	*	*	*	Neo-bladder	PLGA-based scaffold	*	^a^	*	gr 1: cystecomized animals gr 2: weight/age matched human patients	6 months	Occlusion? Histology Immunohistochemstry Voiding intervals Bladder capacity
4	Dorflinger 1985	Research paper	Dogs (Mongrel)	F	gr 1: 17–21 kg gr 2: 22–27 kg^b^	gr 1: 5 gr 2: 5	Neo-bladder	Silicone prosthesis with PGA mesh	65 cc	NA	NA	gr 1: thin prosthesis 2 months preimplant gr 2: thick prosthesis 1 month preimplant	6 months	Occlusion Histology Urogram Mactroscopic evaluation Colony-forming assay
5	Drewa 2007	Research paper	Rats (Wistar)	M	300 g, 6 months	gr 1: 3 gr 2: 3	Urinary conduit 1 ureter	Small intestinal submucosa (SIS), porcine	3 cm 3-layered ^c^	Fibroblast 3T3 2×10∧8 cells in alginate gel	*	gr 1: 2×10∧8 3T3 cells gr 2: unseeded	2 and 4 weeks	Occlusion Histology Pyelogram Macroscopic evaluation
6	Geutjes 2012	Research article	Pigs (Landrace)	F	50 kg	gr 1: 6 gr 2: 4	Urinary conduit 1 ureter	Collagen and Vypro polymer	l = 12 cm, d = 15 mm	UC from 4 cm2 bladder biopsy: 10×10∧6 cells	6 days	gr 1: bladder UC gr 2: unseeded	1 month	Occlusion Histology Immunohistochemistry Loopogram Macroscopic evaluation
7	Kloskowski 2012	Abstract	Rats (Wistar)	*	*	gr 1: 12 gr 2: 2	Urinary conduit 1 ureter	Decellularized aortic arch or PCL scaffold	*	NA	NA	gr 1: aortic arch gr 2: PCL	3 weeks	Occlusion Histology
8	Liao 2013	Research paper	Rabbits (New Zealand White)	M	2,0–2,5 kg	gr 1: 24 gr 2: 6	Urinary conduit 2 ureters	Rabbit bladder acellular matrix (BAM)	l = 4 cm d = 0,8 cm	UC from 4 cm2 bladder biopsy: 80×10∧7 cells	7 days	gr 1: bladder UC gr 2: unseeded	1, 2, 4 and 8 weeks ^d^	Occlusion? Histology Immunohistochemistry

SMC  =  smooth muscle cells, PLGA  =  poly(lactic-co-glycolic acid), PCL  =  polycaprolactone, PGA  =  polyglycolic acid, UC  =  urothelial cells, *  =  not mentioned, N.A.  =  not applicable, ?  =  is implied in the text, but not specifically mentioned, a  =  cells are used, type and amount are not mentioned, b  =  group 3 included only partial cystetomies, as well as 1 dog in group 1 (not included), c  =  diameter of a 12-Fr catheter, d  =  evaluation time point of control group is unclear.

**Table 2:** Scoring of the included studies.

	Reference	Follow-up	Mortality	Adverse effects	Formation of UD	Urothelium formation
1	Basu 2012	84+/−5 days	0%^a^	*	32/32^a^	32/32^b^
2	Bertram 2009	9 months	0%?	none	24/24?^c^	24/24?^c^
3	De Filippo 2009	6 months	0%?	*	*	*
4	Dorflinger 1985	6 months	60%	hydronephrosis (5#), hydroureter (4#), pyonephrosis (2#), inflammation (3#), leakage (2#), infection (1#), ulcers (1#), reflux (2#)	2/10	2/10
5	Drewa 2007	2 or 4 weeks	0%	adhesion (3#), inflammation (4#), leakage (1#), pseudocyst (1#), hydronephrosis (4#), hydroureter (3#)	3/6	1/6
6	Geutjes 2012	1 month	11%	stenosis (3#), leakage (2#), hydroureteronephrosis (all), hydroureter (all)	8/9	6/9
7	Kloskowski 2012	3 weeks	28%^d^	Inflammation (all),	0/14	1/14^e^
8	Liao 2013	1, 2, 4 or 8 weeks	13%^f^	scarring (4#), atresia (4#), hydronephrosis (4#), fistulas (2#), inflammation(2#)^g^	26/30^h^	26/30^h^

*  =  not mentioned, ? =  it is implied that all animals survived and formed a functional conduit with urothelial layers, a  =  all animals were euthanized at indicated time points, animals remained healthy, no explicit mentioning of occlusions, b  =  no mentioning of place of sampling. Unclear whether it covers the entire conduit, c  =  group 4 does not include TE, leaving 24 animals for UD, d  =  deaths were only in aortic arch group, e  =  mentioning of formation of cell layers, not specific on type of cell layer, f  =  all animals died in the experimental group, g =  complications were observed in the control group only, h  =  unclear if this accounts for all animals.

### 4. Methodological quality assessment

All included studies were feasibility studies only, i.e. no comparison was made between the new (tissue engineering) and classical treatment (GI tissue) or any other relevant control group. Therefore, performing a risk of bias-assessment was not possible, and we consequently focused on the quality of the reporting of data and outcomes of the studies (Table 3).

**Table 3:** Quality assessment.

					*Abstracts/Poster*			*Research Articles*				
					Bertram 2009	De Filippo 2009	Kloskowski 2012	Basu 2012	Dorflinger 1985	Drewa 2007	Geutjes 2012	Liao 2013
*Study Design*	**Q1:** Is the animal species described?				yes	no	yes	yes	yes	yes	yes	yes
	**Q2:** Is the specific strain described?				no	no	yes	yes	?	yes	yes	yes
	**Q3:** Is the sex of the animal specified?				no	no	no	yes	yes	yes	yes	yes
	**Q4:** Is the age or weight of the animals specified?				no	no	no	yes	yes	yes	yes	yes
	**Q5:** Is the number of animals specified?				yes	no	yes	yes	yes	yes	yes	yes
	**Q6:** Is the creation of the urinary diversion clearly described?				yes	?	yes	yes	yes	yes	yes	yes
	**Q7:** Is the composition of the tissue-engineered construct clearly described?				no	no	yes	yes	yes	yes	yes	yes
	**Q8:** Are the dimensions of the implanted construct clearly described?				no	no	no	no	yes	yes	yes	yes
	**Q9:** Is the preparation or culture period clearly described?				no	no	no	yes	N.A.	?	yes	yes
*Outcomes*	**Q10:** Are the used outcome measures clearly described?				yes	yes	yes	yes	yes	yes	yes	yes
	**Q11:** Is the follow-up time after implantation clearly described?				yes	yes	yes	yes	yes	yes	yes	?^a^
	**Q12:** Are there any comments on the representativeness of the results?				no	no	no	no	yes	yes	?	no
	**Q13:** Is the location of sampling for histology clearly described?				no	no	no	no	N.A.	yes	yes	no
	**Q14:** Is it explicitly indicated that there were any drop-outs?				no	yes	yes	?	yes	yes	yes	yes
	**Q15:** Is the number of drop-outs described?				N.A.	N.A.	yes	N.A.	yes	N.A.	yes	yes
	**Q16:** Are the reasons for dropping out specified?				N.A.	N.A.	no	N.A.	yes	N.A.	yes	yes
	**Q17:** Is it stated that there were any adverse effects?				yes	?	?	no	yes	yes	yes	yes

?  =  not clearly described in the text, N.A.  =  not applicable, a  =  unknown for the control group.

### 5. Meta-analyses

Meta-analyses were performed for the outcome measures functionality (absence of occlusion) and formation of urothelium in seven studies. One study did not describe the animal species and was therefore excluded [26]. Since appropriate control groups were not included in any of the studies, it was not possible to perform a standard meta-analysis using, for example, odds ratios. We therefore performed a meta-analysis of proportions, more specifically of the number of animals in which a functional construct or urothelium was formed as a proportion of the total number of treated animals. First, exact binomial confidence intervals were calculated for the individual studies. To circumvent continuity corrections (some studies had 0 events), an arcsine transformation of the proportions was carried out for the meta-analyses [21]. Because high heterogeneity was expected, the individual proportions were pooled using a random effects model. Given the low number of studies, a Hartung-Knapp adjustment for random effects models was applied [22]. I^2^ was used as a measure of heterogeneity. The analyses were conducted in R (version 3.0.1; R Core Team 2012), using the metafor package [23], [24]. To explore the potential influence of animal size on the effect, the studies were ranked according to the subgroups “large” (rabbits and larger) and “small” (rats and mice) models in the forest plots. The small group sizes prevented calculation of an overall effect per subgroup and therefore only visually derived tendencies are presented.

## Results and Discussion

### 1. Study inclusion

**Figure 1:**
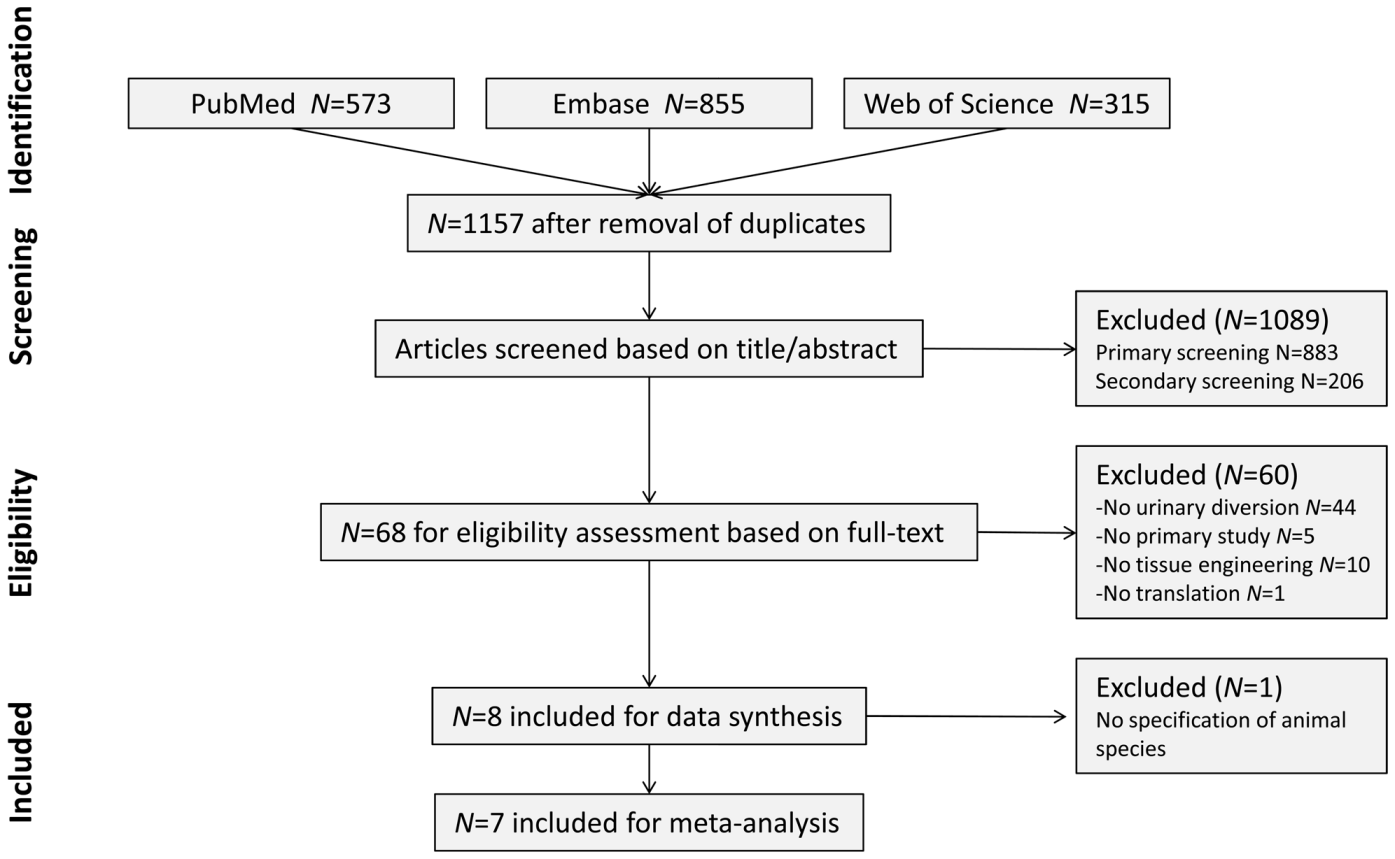
Flow-chart of search and screening process. Primary screening exclusion was performed in End-Note. Criteria included: no urinary diversion, no tissue engineering or reconstruction of ureter or urethra. Secondary inclusion was performed in EROS. Criteria included: no urinary diversion, no tissue engineering, no animal study or no primary study.

Database searches yielded 573 references for PubMed, 855 references for Embase and 315 references for Web of Science (Figure 1). After removal of duplicates and triplicates 1157 references remained. During the primary screening in EndNote, 883 references that did not meet our inclusion criteria were removed. Secondary screening of the remaining 274 references in EROS led to the removal of 206 references. Full text analyses of the remaining 68 references resulted in the inclusion of only 8 studies: two abstracts, one poster and five full-text papers (Figure S1). Screening of the reference lists of these papers and manually identified relevant reviews on the subject did not yield any new references. Thus, the final set included: Geutjes et al. (2012), Basu et al. (2012), Kloskowski et al. (2012), Liao et al. (2013), De Filippo et al. (2009), Bertram et al. (2009), Drewa et al. (2007), and Dørflinger et al. (1985) [11], [25]–[31]. Although the number of published studies on tissue engineering for urinary diversion in animal models appeared to be substantial, only these selected studies applied tissue engineered constructs for urinary diversion at relevant clinical sites in animal models. The recent increase in published papers on this subject is remarkable. Although all relevant databases were explored, we cannot exclude that some data reside within company protected domains. Moreover, studies tend to be published only when results are positive and statistically significant [32]. These two factors may have resulted in an incomplete data set and they may have introduced a publication bias in this systematic review. Due to the limited number of included studies, we were not able to estimate the risk and effect of this publication bias.

### 2. Study characteristics

Analysis of study characteristics revealed extraordinary diversity (Table 1). Various animal species, including pigs, minipigs, dogs, rats and rabbits, were used. They were implanted with constructs of either biodegradable polymers (poly(lactic-*co*-glycolic acid) (PLGA), polyglycolic acid (PGA) or collagen) or decellularized material (small intestinal submucosa (SIS) and bladder acellular matrix (BAM)). Kloskowski et al., used a biodegradable polymer polycaprolactone (PCL) and decellularized aortic arches. Although three different approaches for urinary diversion are known (urinary conduit, abdominal pouch and neobladder), the included studies only created urinary conduits (5 studies) or neobladders (3 studies). An animal model for an abdominal pouch was not described.

There are three different study designs within the pool of included studies: studies that use cellular or acellular scaffolds and studies that compare these two constructs (Table 1 and 4). The most frequently used study design was the comparison between cellular and acellular constructs (4 studies), investigating whether (pre-)seeding of the biomaterial resulted in a superior outcome. These studies generally isolated their cells from a bladder biopsy, although alternative sources like adipose tissue, peripheral blood and cell lines were investigated (Basu et al., Drewa et al., Geutjes et al. and Liao et al.). The other studies compared different materials (Kloskowski et al.), cell concentrations (Bertram et al.) or different experimental designs (Dørflinger et al.). Filippo et al. compared the behavior of a cell-seeded PLGA-based scaffold in cystectomized animals with its behavior in children enrolled in a Phase II trial for bladder augmentation. This diversity in animal species, biomaterial or study design complicates the interpretation of the results of this systematic review.

**Table 4:** Cellular vs. Acellular.

	Acellular	Cellular
Basu et al 2012	8/8	24/24
Drewa et al 2007	2/3	1/3
Geutjes et al 2012*	4/4	5/6
Liao et al 2013	4/6	24/24

Amount of functional conduits formed in comparative studies with cellular and acellular groups. * was tabulated after correspondence with the first author.

Since tissue engineering for urinary diversion is a relatively new area of research, the main focus of the studies was to determine the feasibility of implantation and subsequent behavior of the designed constructs. Follow-up time was less than a year in all cases, whereas constructs need to be functional throughout the remainder of a patients' life time. Stoma complications can occur after several years in patients, and short follow-up in animals will not provide evidence on late complications. This indicates the necessity of appropriate control groups with the classical techniques and longer follow-up for at least 1 year.

### 3. Quality assessment

The lack of control groups precluded a risk of bias-analysis. We therefore focused on the reporting quality of the studies (Table 3). Our specific interests were the animal characteristics, the composition, dimensions and preparations of the construct, the representativeness of the results and the adequate reporting of drop-outs.

The reporting was relatively poor in the abstracts compared to the full-text papers. Many abstracts omitted important details that are essential to compare different studies, including animal species, strain, number and sex, type of tissue engineered scaffolds, description of the composition, dimensions and preparations of the construct (Q1-Q9). This might have been partly the consequence of the word and space limitation for abstracts.

All included studies appropriately described the predefined outcome measures and stated at which time evaluation took place. Only two studies commented on the representativeness of the figures for the overall study outcomes (Dørflinger et al. and Drewa et al.) (Q10-Q13). In total, three papers were complete in their reporting of the study design (Drewa et al., Geutjes et al., and Liao et al.).

Although guidelines for standardized reporting of animal experimentation have been described, these have not yet been generally accepted [33], [34]. We observed that in some studies, especially in the abstracts, these guidelines were not implemented. It is crucial to further improve methodological reporting to aid future research, even in abstracts.

### 4. Data synthesis

To determine the efficacy of the tissue engineered constructs, we focused on the outcome measures: formation of a functional conduit or reservoir and the formation of urothelium in the regenerated tissue, evidenced by histology (Table 2). Some studies performed additional analyses, including immunohistochemistry, urograms or pyelograms (Table 1), but these were not considered here. Because constructs can only be functional in the absence of major complications (including mortality), we first looked at the survival of the animals and adverse effects. Survival of the animals was regarded as the first indication for the safety of a construct and represented a condition for the efficacy of a construct. Secondly, since urine needs to exit the body adequately, the formation of a reservoir or conduit with a urothelial lining was deemed essential.

#### 4.1 Adverse effects and mortality

Experience shows that even in some situations animals might die of unrelated causes, with more likelihood in larger experimental groups. A clear and detailed description of the drop-outs will increase the credibility of the study. Surprisingly, two studies did not explicitly report on the mortality rate, but the studies suggest that all animals survived the procedure in good health (Basu et al, and Bertram et al.) They imply that a functional urinary conduit with urothelial linings is formed in all animals without any adverse effects (Table 2 and Table 3, Q14-Q17). Such a successful score was not described in any of the other studies, which raises the possibility that the success rate was overestimated. The reported mortality of the other studies ranged between 60% in the pioneering study in 1985 (Dørflinger et al.) to 13% in the most recently published paper (Liao et al.), suggesting the application of improved constructs or improved surgical techniques over the years. Geutjes et al. is the only study that reports on both related and unrelated deaths.

All studies report on inflammation of the construct and the surrounding tissues, although this does not necessarily constitute a negative effect. Some degree of inflammation may guide tissue regeneration, remodeling and the formation of blood vessels. The formation of a vasculature structure in large constructs still remains a major problem in tissue engineering [35], [36]. Other common adverse effects found in the majority of the studies were hydronephrosis and hydroureters, prevention of which remains the biggest challenge for tissue engineers. The designed constructs were not able to control urinary pressure, leading to reflux to the kidney and the aforementioned conditions.

The formation of stones in urinary diversions is one of the complications in using GI tissue. Remarkably, only one study, performed in pigs reported the formation of calcifications (Geutjes et al.). The absence of stones, particularly in the included rabbit study, is unexpected (Liao et al.). Implantation of a tissue engineered patch in the bladder wall of rabbits resulted in a high incidence of stone formation and encrustation, indicating that this animal model in particular is prone to develop stones [37], [38]. Although a different biomaterial was used in a different setting, no stones were formed up until two months in the study performed by Liao et al. In humans, it takes months to years to develop urinary stones and perhaps the follow-up time in these studies is too short (<1 year) to detect stones [39]. Obviously, due to the difference in diet composition, flow speed, composition and pH of urine in different species, urinary stones might not develop in some animals. Long-term follow-up is necessary to exclude encrustation or stone formation.

#### 4.2 Efficacy

**Figure 2:**
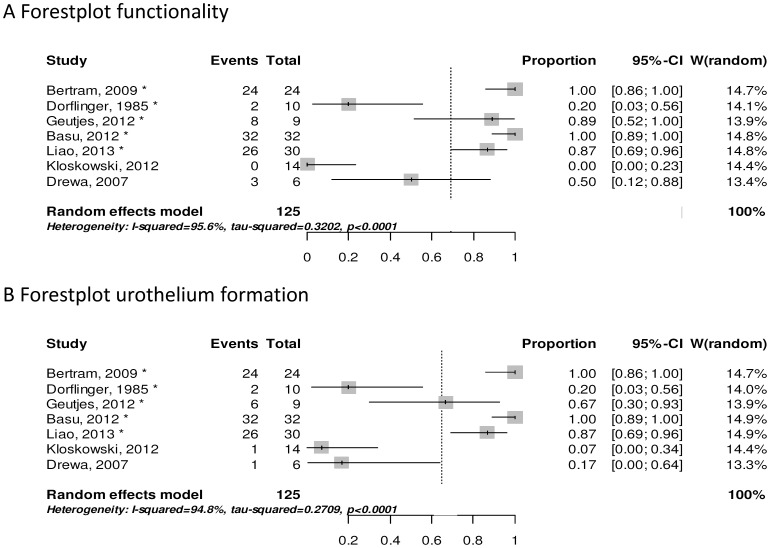
Forestplots for functionality (A) and urothelium formation (B). Forest plot of 7 studies. Filippo et al. was excluded for both meta-analyses since the study does not report on the type of animal, functionality and urothelium formation. * indicates studies with large animal species.

We conducted meta-analyses for the outcome measures functionality and urothelium formation (Figures 2A and B). The aim of these meta-analyses was not to obtain a precise point estimate, but rather to get an impression of the quantitative relations between the results of the individual studies and to detect trends [40]. The heterogeneity between the studies was very high (I^2^ = 96% for functionality and I^2^ = 95% for urothelium formation). Although this did not justify pooling of the results, nevertheless, overall proportions were calculated for functionality and urothelium formation (69% [CI: 18–100%] and 65% [CI: 17–98%], respectively). Due to the large heterogeneity and small number of studies, these overall proportions should be interpreted with caution.To prevent misinterpretations, overall proportions are only shown as dotted vertical lines in the forest plots.

Successful formation of functional conduits with full urothelial linings, the primary goal of all studies, varied between the studies. Only small numbers of functional conduits and urothelial coverage were observed by Dørflinger et al., Drewa et al. and Kloskowski et al. In contrast, Liao et al. showed that in 4/5 cases a functional conduit was formed with urothelial linings. The large heterogeneity and small number of studies complicates identification of the underlying cause of the different results. We can only speculate whether these were the consequence of the biomaterial, the animal model or confounding factors.

#### 4.3 Animal models

The small number and heterogeneity of the studies did not permit subgroup analysis for different animal species. However, the forest plots (Figures 2A and B) from the meta-analyses suggested a tendency towards better results in large animal models compared to small animals. Although we cannot exclude the effect of confounding factors, this supports the idea that future research should focus on larger animals. Larger animals have a more similar anatomy to the human body than, for example, rodents, and allow for evaluation at clinical relevant sites with constructs of a comparable size. Moreover, surgical techniques and materials are more comparable to the human setting, therefore mimicking the human situation as closely as possible. In 1985, the first report on the use of a construct to tissue engineer a urinary diversion as a replacement for GI tissue in large animals was published (Dørflinger et al.). Even though the mortality rate was high, a functional reservoir could be formed showing the feasibility of this approach. In later studies small animals were used (Kloskowski et al. and Drewa et al.) but the success rate in these studies was low. This might be explained by the surgical challenge with a higher risk of complications in smaller animals.

The extraordinary heterogeneity in animal studies has previously been reported by Roosen et al. [41], who reviewed different animal models for the classical types of urinary diversion using GI tissue. A uniform conclusion regarding the most suitable animal model could not be drawn and the author advised to view the results with caution, when translating these for clinical implementation.

#### 4.4 Cellular and acellular scaffolds

The studies that compared acellular and cellular scaffolds investigated the effect of (pre-) seeding on tissue regeneration (Table 1 and 4). Basu et al., Liao et al. and Geutjes et al. expanded autologous cells from a biopsy. Basu et al. stated that (pre-)seeding of the scaffolds provided an additional advantage, although this was not substantiated. The same conclusion was drawn by Liao et al., but here the authors clearly report that the drop-outs and adverse effects in the acellular group were higher. Geutjes et al. observed no difference between cellular and acellular constructs.

In the study by Drewa et al., scaffolds were seeded with 3T3 fibroblast from mouse origin, followed by implantation in rats. Not surprisingly, this resulted in an excessive inflammatory response and consequently a better outcome for the unseeded group.

The presence of a cellular component can trigger M1 macrophages, resulting in fibroblast deposition. In contrast, acellular scaffolds activate the M2 macrophages, which leads to reconstruction and regeneration of tissues [35]. Some researchers have reported that scaffold (pre-)seeding provides an additional advantage for tissue regeneration, since regeneration is less dependent on cellular in-growth [42], [43]. Since the effect pre-seeding is outside the scope of this systematic review, a meta-analyses was not performed. Therefore, based on the available information, it is unclear whether (pre-)seeding of the scaffolds provides an advantage for tissue reconstruction in urinary diversions.

#### 4.5 Concluding remarks

Based on this systematic review the most adequate animal model for urinary diversion is still undefined. Only a limited amount of studies could be identified despite the comprehensive search strategy, all showing large heterogeneity. It appears that the predictive value of a particular animal model was not a decisive factor in the studies performed. Nevertheless, the forest-plots suggested a trend towards successful experimentation in larger animal models, supporting the idea that future research should focus on the evaluation of constructs in larger animal models which more closely mimic the human body than small (rodent) animal models. Bladder augmentations and urethral replacements have been successful in dogs [44]–[47]. Although the results of the earliest study were disappointing (Dorflinger) this might be explained by the tissue engineering strategy. Use of a more sophisticated tissue engineering approach did lead to satisfactory results (Bertram). In view of the former, evaluation of urinary diversion constructs in dogs might be a valid alternative.

Despite the limited amount of data available on this subject, a phase I clinical trial was initiated for implantation of a (pre-)seeded tissue-engineered urinary conduit in cystectomized patients [48]. Whether it is acceptable to expose patients to a new device which has not been investigated in a long term and multicenter animal study remains a matter of debate. Critics of animal experimentation might say that it is difficult to find a representative animal model to improve clinical translation. In order to improve translational practice, a re-evaluation of preclinical practice is warranted, in which systematic reviews and meta-analyses of animal studies can provide a valuable tool [49].

This systematic review focused on the identification of an appropriate animal model to investigate tissue engineering for urinary diversion. Remarkably, only feasibility studies were identified, which are necessary to evaluate potentially valuable techniques. To obtain a more thorough estimation of the feasibility and applicability, a large variety of approaches should be investigated and should include different biomaterials, growth factors and large animal models. Evaluation of the functionality and advantages of newly developed constructs should include appropriate controls and evaluate long-term effects and outcomes to investigate safety and efficacy of these constructs. Moreover, standardized assessment methods are desirable. The ultimate control would be the use of GI tissue, the golden standard technique currently used in the clinic. Assessment of these preclinical experiments should focus on both functionality and tissue regeneration, using urodynamic measurements and histological evaluations. To continue to clinically translatable studies, standardization of (large) controlled and comparative studies with adequate methodological reporting is required as laid down in existing guidelines [33], [34].

Animal experimentation remains subject to ethical debate and -among others- for animal welfare reasons it is therefore essential that research is properly conducted and reported. Nevertheless, the reporting of the methodology and representativeness of results was rather poor in the reviewed studies. Improvements in this area are urgently needed for ethical, scientific and economic reasons, and will allow researchers to repeat studies reliably and make an unbiased decision on the most correct animal model for their future experiments. The scientific community should adopt the existing guidelines for standardized reporting of animal experimentation and implement them in journal author guidelines, which should be enforced by editors, to improve standardized reporting in full-text articles. This will aid in good quality research with more relevant output for the clinic and reduction of the use of animals in biomedical research.

## Supporting Information

Figure S1 **Inclusion and exclusion of papers during secondary and full-text screening in EROS.** After primary screening 274 papers were analyzed in EROS, resulting in the inclusion of 8 papers. Papers were excluded which were without urinary diversion, tissue engineering or animals or when they were reviews. For one study, we did not have the resources for translation.
(PDF)

Table S1 **Complete search strategy for PubMed, EMBASE and Web of Science.** MeSH and EMTREE terms were used when possible and combined with free-text words from title or abstract ([tiab] or.ti,ab.).
(PSD)

Checklist S1 **PRISMA checklist.**
(DOC)

## References

[1] National Cancer Institute (2010) What you need to know about bladder cancer. NIH publiciation No. 10–1559.

[2] Pannek J, Senge T (1998) History of urinary diversion. Urol Int 60: 1–10. 10.1159/000030195 9519414

[3] Hautmann RE (2003) Urinary diversion: ileal conduit to neobladder. J Urol 169: 834–842. 12576795 10.1097/01.ju.0000029010.97686.eb

[4] Kaefer M, Hendren WH, Bauer SB, Goldenblatt P, Peters CA, et al (1998) Reservoir calculi: a comparison of reservoirs constructed from stomach and other enteric segments. J Urol 160: 2187–2190. 9817364

[5] Hautmann RE, Hautmann SH, Hautmann O (2011) Complications associated with urinary diversion. Nat Rev Urol 8: 667–677. 22045349 10.1038/nrurol.2011.147

[6] Nieuwenhuizen JA, de Vries RR, Bex A, van der Poel HG, Meinhardt W, et al (2008) Urinary diversions after cystectomy: the association of clinical factors, complications and functional results of four different diversions. Eur Urol 53: 834–842. 17904276 10.1016/j.eururo.2007.09.008

[7] Drewa T, Adamowicz J, Sharma A (2012) Tissue Engineering for the oncologic urinary bladder. Nat Rev Urol 9: 561–572. 22907387 10.1038/nrurol.2012.158

[8] Kim BS, Baez CE, Atala A (2000) Biomaterials for tissue engineering. World J Urol 18: 2–9. 10766037 10.1007/s003450050002

[9] Beiko DT, Knudsen BE, Watterson JD, Cadieux PA, Reid G, et al (2004) Urinary tract biomaterials. J Urol 171: 2438–2444. 15126872 10.1097/01.ju.0000125001.56045.6c

[10] Drewa T, Sir J, Czajkowski R, Wozniak A (2006) Scaffold seeded with cells is essential in urothelium regeneration and tissue remodeling in vivo after bladder augmentation using in vitro engineered graft. Transplant Proc 38: 133–135. 16504684 10.1016/j.transproceed.2005.11.086

[11] Geutjes P, Roelofs L, Hoogenkamp H, Walraven M, Kortmann B, et al (2012) Tissue engineered tubular construct for urinary diversion in a preclinical porcine model. J Urol 188: 653–660. 22704444 10.1016/j.juro.2012.03.119

[12] Raya-Rivera A, Esquiliano DR, Yoo JJ, Lopez-Bayghen E, Soker S, et al (2011) Tissue-engineered autologous urethras for patients who need reconstruction: an observational study. Lancet 377: 1175–1182. 21388673 10.1016/S0140-6736(10)62354-9 PMC4005887

[13] Atala A, Bauer SB, Soker S, Yoo JJ, Retik AB (2006) Tissue-engineered autologous bladders for patients needing cystoplasty. Lancet 367: 1241–1246. 16631879 10.1016/S0140-6736(06)68438-9

[14] Wood MW, Hart LA (2007) Selectin appropriate animal models and strains: making the best use of research, information and outreach. AATEX 14: 303–306.

[15] Nordgren A (2004) Moral imagination in tissue engineering research on animal models. Biomaterials 25: 1723–1734. 14697873 10.1016/s0142-9612(03)00506-4

[16] De Vries RB, Buma P, Leenaars M, Ritskes-Hoitenga M, Gordijn B (2012) Reducing the number of laboratory animals used in tissue engineering research by restricting the variety of animal models. Articular cartilage tissue engineering as a case study. Tissue Eng Part B Rev 18: 427–435. 22571623 10.1089/ten.TEB.2012.0059

[17] Leenaars M, Hooijmans CR, van Veggel N, ter Riet G, Leeflang M, et al (2012) A step-by-step guide to systematically identify all relevant animal studies. Lab Anim 46: 24–31. 22037056 10.1258/la.2011.011087 PMC3265183

[18] Hooijmans CR, Tillema A, Leenaars M, Ritskes-Hoitenga M (2010) Enhancing search efficiency by means of a search filter for finding all studies on animal experimentation in PubMed. Lab Anim 44: 170–175. 20551243 10.1258/la.2010.009117 PMC3104815

[19] De Vries RB, Hooijmans CR, Tillema A, Leenaars M, Ritskes-Hoitinga M (2011) A search filter for increasing the retrieval of animal studies in Embase. Lab Anim 45: 168–270. 10.1258/la.2011.011056 PMC3175570 21890653

[20] Lee YS, Cho SY, Kim HW, Kang SH, Kim HY, et al (2009) Preliminary study of tissue-engineered ileal conduit using poly (e-caprolactone) (PCL) nano-sheet seeded with muscle-derived stem cells. . Korean J Urol. 50: 282–287.

[21] Rücker G, Schwarzer G, Carpenter J, Olkin I (2009) Why add anything to nothing? The arcsine difference as a measure of treatment effect in meta-analysis with zero cells. Stat Med 28: 721–728. 19072749 10.1002/sim.3511

[22] Knapp G, Hartung J (2003) Improved tests for a random effects meta-regression with a single covariate. Stat Med 22: 2693–2710. 12939780 10.1002/sim.1482

[23] R Core Team (2012) R: a language and environment for statistical computing. R foundation for statistical computing. ISBN 3-900051-07-0.

[24] Viechtbauer W (2010) Conducting meta-analyses in R with the metafor package. J Stat Soft 36: 1–48.

[25] Liao W, Yang S, Song C, Li Y, Meng L, et al (2013) Tissue-engineered tubular graft for urinary diversion after radical cystectomy in rabbits. J Surg Res 182: 185–191. 23140788 10.1016/j.jss.2012.10.024

[26] De Filippo R, Bertram TA, Jayo MJ, Seltzer E (2009) Adaptive regulation of regenerated bladder size after implantation with tengion neo-bladder augment™ early clinical outcomes and preclinical evidence. J Urol 181: 267.

[27] Drewa T (2007) The artificial conduit for urinary diversion in rats: a preliminary study. Transplant Proc 39: 1647–1651. 17580209 10.1016/j.transproceed.2007.02.092

[28] Dørflinger T, Frimodt-Møller PC, England DM, Madsen PO, Bruskewitz R (1985) Prosthetic urinary bladder implantation to facilitate bladder regeneration. Neurourol Urodyn 4: 47–59.

[29] Basu J, Jayo MJ, Ilagan RM, Guthrie KI, Sangha N, et al (2010) Regeneration of native-like neo-urinary tissue from non-bladder cell sources. Tissue Eng Part A 18: 1025–1034. 10.1089/ten.TEA.2011.0569 22136657

[30] Kloskowski T, Jundzill A, Gurtowska N, Olkowska J, Kowalczuk T, et al (2012) Urinary conduit construction using tissue engineering. J Regen Med Tissue Eng 6: 23.

[31] Bertram TA, Christ GJ, Andersson KE, Aboushwareb T, Fuellhase C, et al (2009) Pharmacologic response of regenerated bladders in a preclinical model. FASEB J 23: 291.

[32] Knight J (2003) Negative results: Null and void. Nature 422: 554–555. 12686968 10.1038/422554a

[33] Kilkenny C, Browne WJ, Cuthill IC, Emerson M, Altman DG (2010) Improving bioscience research reporting: the ARRIVE guidelines for reporting on animal research. PLoS Biol 8: e1000412. 20613859 10.1371/journal.pbio.1000412 PMC2893951

[34] Hooijmans C, Leenaars M, Ritskes-Hoitenga M (2010) A gold standard publication checklist to improve the quality of animal studies, to fully integrate the Three Rs, and to make systematic reviews more feasible. Altern Lab Anim 38: 167–182. 20507187 10.1177/026119291003800208

[35] Brown BN, Valentin JE, Stewart-Akers AM, McCabe GP, Badylak SF (2009) Macrophage phenotype and remodeling outcomes in response to biologic scaffolds with and without cellular component. Biomaterials 30: 1482–1491. 19121538 10.1016/j.biomaterials.2008.11.040 PMC2805023

[36] Badylak SF, Valentin JE, Ravindra AK, McCabe GP, Stewars-Akers AM (2008) Macrophage phenotype as a determinant of biologic scaffold remodeling. Tissue Eng Part A 14: 1835–1842. 18950271 10.1089/ten.tea.2007.0264

[37] Nuininga JE, van Moerkerk H, Hanssen A, Hulsbergen CA, Oosterwijk-Wakka J, et al (2004) A rabbit model to tissue engineer the bladder. Biomaterials 25: 1657–1661. 14697867 10.1016/s0142-9612(03)00519-2

[38] Grover PK, Ryall RL (1994) Urate and calcium oxalate stones: from repute to rhetoric to reality. Miner Electrolyte Metab 20: 361–370. 7783698

[39] Evans K, Costabile RA (2005) Time to development of symptomatic urinary calculi in a high risk environment. J.Urol 173: 858–861. 15711293 10.1097/01.ju.0000152578.07262.1c

[40] Vesterinen HM, Sena ES, Egan KJ, Hirst TC, Churolov L, et al (2014) Meta-analyis of data from animal studies: a practical guide. J Neurosci Methods 221: 92–102. 24099992 10.1016/j.jneumeth.2013.09.010

[41] Roosen A, Woodhouse CR, Wood DN, Stief CG, McDougal WS, et al (2011) Animal models in urinary diversion. BJU Int 109: 6–23. 21917109 10.1111/j.1464-410X.2011.10494.x

[42] De Filippo RE, Yoo JJ, Atala A (2002) Urethral replacement using cell seeded tabularized collagen matrices. J Urol 168: 1789–1792. 12352360 10.1097/01.ju.0000027662.69103.72

[43] Zhang Y, Kropp BP, Lin HK, Cowan R, Cheng EY (2004) Bladder regeneration with cell-seeded small intestinal submucosa. Tissue Eng 10: 181–187. 15009944 10.1089/107632704322791835

[44] Sievert KD, Fandel T, Wefer J, Gleason CA, Nunes L, et al (2006) Collagen I:III ratio in canine heterologous bladder acellular matrix grafts. World J Urol 24: 101–109. 16474953 10.1007/s00345-006-0052-8

[45] Probst M, Piechota HJ, Dahiya R, Tanagho EA (2000) Homologous bladder augmentation in dog with the bladder acellular matrix graft. BJU Int 85: 362–371. 10671897 10.1046/j.1464-410x.2000.00442.x

[46] Orabi H, Aboushwareb T, Zhang Y, Yoo JJ, Atala A (2013) Cell-seeded tabularized scaffolds for reconstruction of long urethral defects: a preclinical study. Eur Urol 63: 531–538. 22877501 10.1016/j.eururo.2012.07.041 PMC3554849

[47] Roth CC, Mondalek FG, Kibar Y, Ashley RA, Bell CH, et al (2011) Bladder regeneration in a canine model using hyaluronic acid-poly(lactic-co-glycolic-acid) nanoparticle modified porcine small intestinal submucosa. BJU Int 108: 148–155. 20942834 10.1111/j.1464-410X.2010.09757.x

[48] ClinicalTrials.gov (2014) Incontinent urinary diversion using an autologous neo-urinary conduit. Identifier NCT 01087697.

[49] Hooijmans CR, Ritskes-Hoitinga M (2013) Progress in using systematic reviews of animal studies to improve translational research. PLoS Med 10: e1001482. 23874162 10.1371/journal.pmed.1001482 PMC3712909

