# Correction: Tissue Engineering in Animal Models for Urinary Diversion: A Systematic Review

**DOI:** 10.1371/journal.pone.0105484

**Published:** 2014-08-06

**Authors:** The PLOS ONE Staff

## Notice of Republication

This article was republished on July 25, 2014, to correct the spelling of the 4^th^ author’s name within the byline and citation. Please download this article again to view the corrected version. The originally published, uncorrected article and the republished, corrected article are provided here for reference.

## Supporting Information

File S1 Originally published, uncorrected article
(PDF)

File S2 Republished, corrected article
(PDF)
