# Supplementary figures and images for: Tissue Engineering in Animal Models for Urinary Diversion: A Systematic Review
Source: PLoS One. 2014 Jun 25;9(6):e98734. doi: 10.1371/journal.pone.0098734 (PMC4070912; doi:10.1371/journal.pone.0098734)

### Inclusion and exclusion in EROS

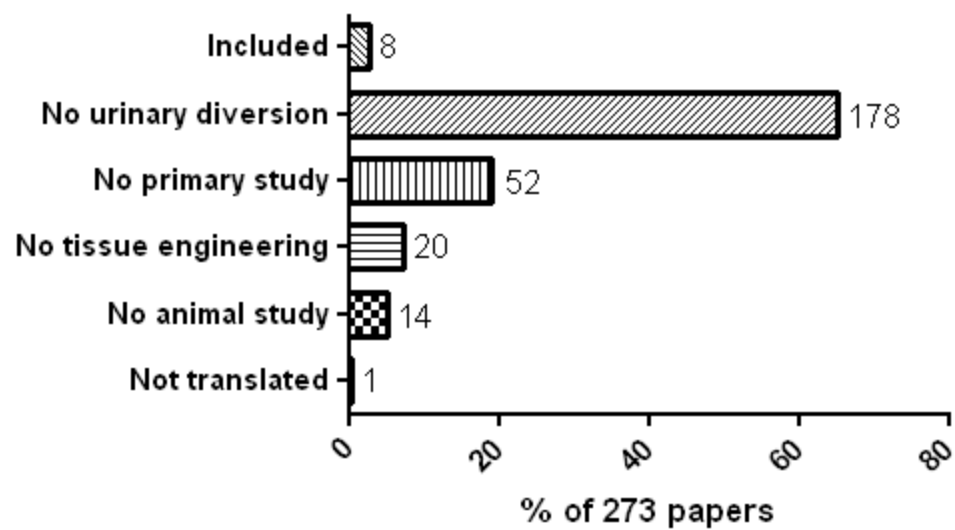

Supplement: Figure S1 — Inclusion and exclusion of papers during secondary and full-text screening in EROS. After primary screening 274 papers were analyzed in EROS, resulting in the inclusion of 8 papers. Papers were excluded which were without urinary diversion, tissue engineering or animals or when they were reviews. For one study, we did not have the resources for translation. (PDF) [file pone.0098734.s001.pdf]
